# Three-dimensional printing of ice structures via evaporative cooling in vacuum

**DOI:** 10.1073/pnas.2608173123

**Published:** 2026-09-02

**Authors:** Menno Demmenie, Stefan Kooij, Daniel Bonn

**Affiliations:** ^a^ Van der Waals-Zeeman Institute, Institute of Physics, University of Amsterdam, Amsterdam 1098 XH, The Netherlands

**Keywords:** ice crystallization, 3D printing, evaporative cooling, phase-change materials, vacuum-assisted processing

## Abstract

We demonstrate an approach to three-dimensional (3D) printing of ice structures by exploiting evaporative cooling. A micrometer-sized water jet is used to 3D print inside a vacuum chamber. The reduced ambient pressure leads to rapid evaporation of the extruded water, extracting latent heat, and quickly cooling the water well below 0 °C. Once deposited, the water freezes almost instantaneously into stable ice structures. We achieved high-fidelity printing via two distinct deposition techniques: layer-by-layer deposition for intricate 3D structures (e.g., a Christmas tree), and support-free mid-air printing for upright profiles (e.g., an in profile face), all achieved without cryogenic infrastructure, supporting materials, or external refrigeration. This approach directly visualizes fundamental thermodynamic principles—latent heat, evaporative cooling, and pressure-dependent phase transitions—while offering a relatively simple and scalable platform for ice-templated microfluidics and tissue engineering, or even extraterrestrial 3D printing.

## Significance

Three-dimensional printing of ice offers a unique route to manufacturing complex structures, yet it typically requires expensive, energy-intensive cryogenic infrastructure. We demonstrate a simplified approach that allows for printing at room temperature by utilizing the rapid evaporative cooling of water in a vacuum. This technique produces stable, free-standing ice geometries without chemical additives. Such ice structures hold significant potential as sacrificial templates: once embedded in other materials, ice could be melted away to create residue-free voids. This capability offers a promising pathway to address fabrication challenges in creating vascular networks for tissue engineering and complex channel systems for microfluidics. Beyond fabrication, this accessible technique suggests a strategy for in situ resource utilization on planetary bodies like the Moon or Mars.

Three-dimensional (3D) printing of ice has attracted recent interest as a route to complex, intricate scaffolds for tissue engineering, microfluidic devices, and casting templates (1–7). Conventional approaches rely on cryogenic refrigeration (using liquid nitrogen or helium) or substrate cooling to stabilize solid water, which requires specialized equipment and infrastructure, and often entails significant operational costs. Here, we introduce a fundamentally different technique by exploiting the rapid evaporative cooling of water under reduced pressure to enable spontaneous freezing of water deposited by a micron-sized jet, requiring only a vacuum pump and a modified commercial 3D printer. We successfully demonstrate two distinct fabrication modes: conventional layer-by-layer deposition and support-free mid-air printing. While both techniques were previously pioneered using drop-on-demand dispensing on a cold surface (1, 2), our vacuum-based setup executes them approximately 100 times faster. Also, conventional cold-surface printing suffers from diminishing speeds as the object grows taller, since the latent heat must be conducted away through the increasingly thick, insulating ice layer below. This fundamental thermal bottleneck severely limits the upscaling of such techniques to larger geometries. Evaporative cooling, however, completely bypasses this diffusion limit by extracting heat directly from the liquid surface. Only a small fraction of the water needs to evaporate to induce freezing: the printed volume does not visibly diminish before solidification. Once formed, the ice does undergo some sublimation, since printing is performed at room temperature and neither the printer nor the build plate is actively cooled. However, this sublimation is limited over the timescale of printing, and the printed structures remain intact for a surprisingly long time, especially considering their thin and delicate geometries.

Interestingly, the low ambient pressures on the Moon ($10^{-12}$ to $10^{-13}$ mbar) and Mars (6 mbar) are within the range required for this freezing mechanism (8, 9). This presents a compelling opportunity for in situ resource utilization (ISRU), directly addressing the strict requirements and current engineering challenges of extraterrestrial additive manufacturing (10, 11). Under these conditions, water-based or particle-laden jets could be used to directly print structures in these environments without additional cooling systems (12). The physical viability of utilizing 3D-printed ice for radiation shielding and protective habitats has already been established by prior proposals, including the winning design of the NASA 3D-Printed Habitat Challenge (13). Furthermore, ice mixed with sand or regolith—similar to natural permafrost—can form a strong composite material (14, 15), especially when 3D infill geometries are optimized to minimize water use.

## 1. Results

**Fig. 1.**
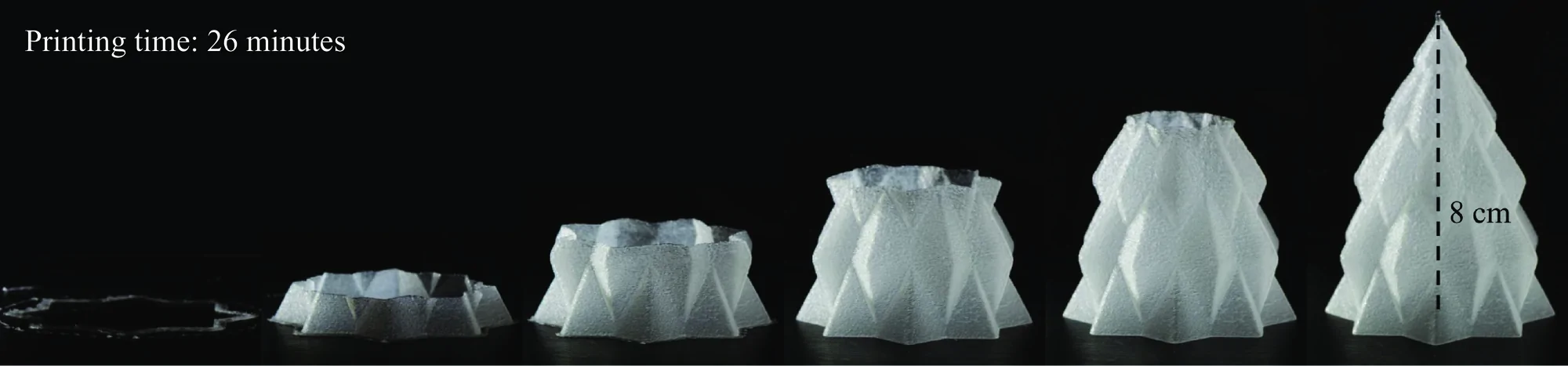
Time-lapse of the 3D-printing process leading up to an ice Christmas tree grown layer by layer. Total construction time was 26 min at 2 mbar.

Fig. 1 shows a time-lapse sequence of the printing process; the complete tree required 26 min of printing time. The full printing process is captured in the Movie S1, while its melting sequence when the vacuum is removed is provided in Movie S2. The Christmas tree structure was printed in spiral mode to a height of approximately 8 cm, with a base diameter of 6 cm and fine detail features (branches, asymmetries) reproduced from the 3D model. The method is remarkably simple: it requires no external cooling coils, no cryogenic gas infrastructure, and no supporting sacrificial material to stabilize the structure.

In order to 3D print ice without additional cooling, the absorption of latent heat during evaporation must be sufficient to cool the remaining liquid to the desired temperature below 0 °C. This energy balance is governed by

[1] $$\begin{array}{c} m_{\text{liq}}\cdot c_{p}\frac{\mathrm{dT}}{\mathrm{dt}}=-L_{v}\frac{dm_{\text{evap}}}{\mathrm{dt}}, \end{array}$$

where $m_{\mathrm{liq}}$ is the liquid mass of the droplet, $m_{\text{evap}}$ is the evaporated mass $c_{p}\approx 4.2$ kJ (kg^−1^ K^−1^) is the specific heat capacity, $L_{v}\approx 2.45$ MJ kg^−1^ is the latent heat of vaporization, and $dm_{\text{evap}}/dt$ is the mass evaporation rate. For a jet of radius r=8μm and surface area A≈2πrL (where L is travel distance), evaporation of less than 5% of the jet mass per centimeter of travel can already account for the observed temperature drop.

**Fig. 2.**
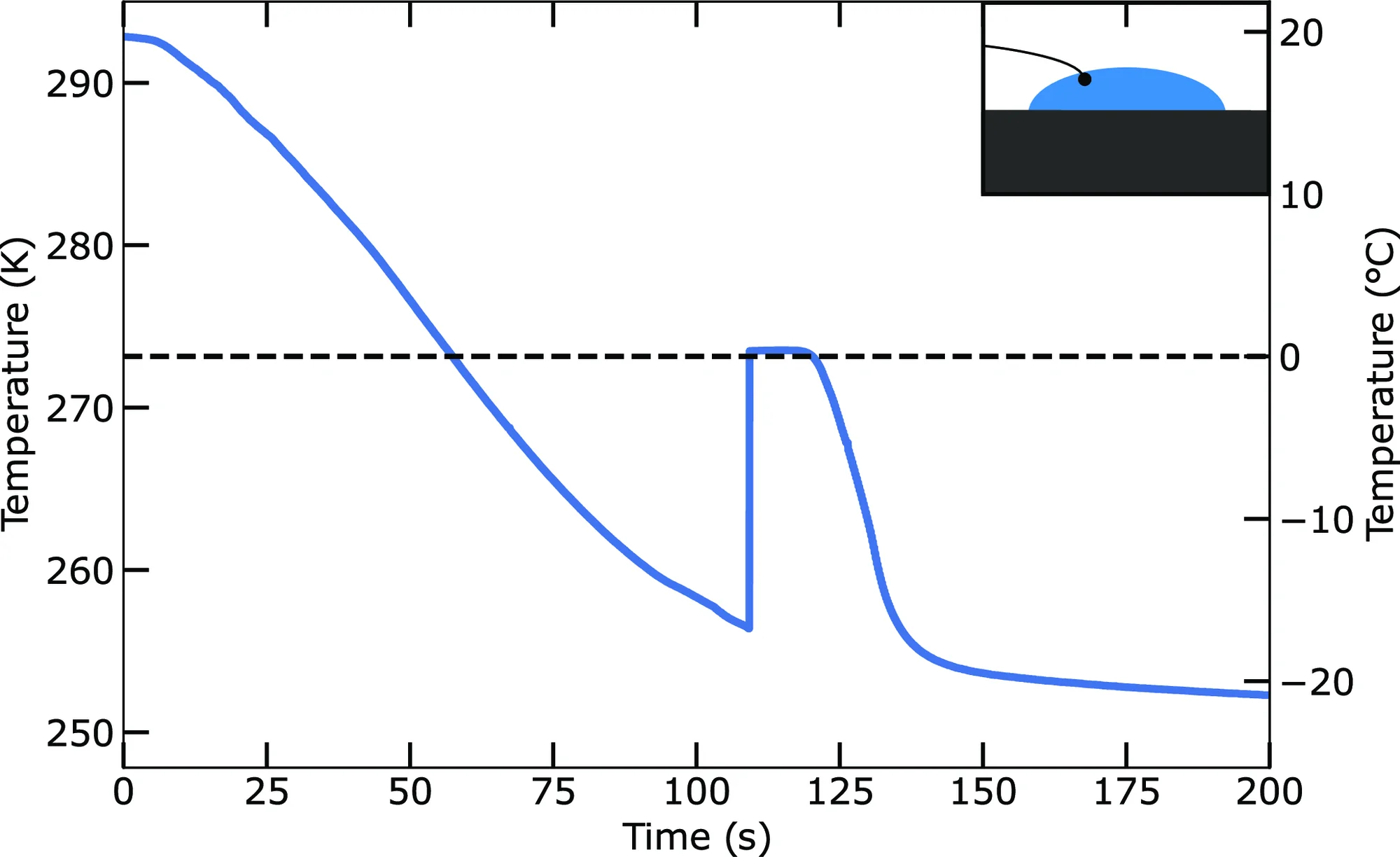
Evaporative cooling of a sessile drop under similar vacuum conditions (2 to 3 mbar) as used for 3D printing. A macroscopic droplet of 3 mm in radius was chosen to enable contact thermometry (Pico TC-08, using a 1 ms sampling interval), serving as a proxy for the smaller 3D-printing droplets. Recalescence occurs at 110 s; the droplet freezes rapidly from an undercooled state, releasing latent heat that drives a sudden temperature rise to $0^{{}^{\circ}}\;\;\text{C}$ (dotted line). The *Inset* shows the experimental schematic.

To understand this process, we perform a simple reference experiment in which we freeze a mm-sized drop in the same vacuum setup. A simple scaling argument shows that the observed freezing distance of the 16 μm jet is fully consistent with the measured cooling of a millimetric drop in the same vacuum environment. We performed contact thermometry experiments on a sessile water drop of radius R=3mm (Fig. 2) which reveals evaporative cooling from room temperature to zero degrees over a timescale of ≈50 s, corresponding to a characteristic cooling rate ${\dot{T}}_{\text{drop}}\approx 0.4\;\;\text{K/s}$. For our spherical cap of 3 mm radius with a contact angle of 50 °, the surface-to-volume ratio which sets the evaporation rate is ≈1,600 m^−1^. For the liquid jet of 5 cm length and jet diameter 16 μm the surface to volume ratio is about a factor of 150 larger than for the millimetric drop. If the evaporative cooling rate scales linearly with the surface to volume ratio, the jet therefore requires only 0.3 s to reach subzero temperatures. This estimate is conservative, as it neglects the difference between the way the drop and jet experiments are performed: the drop starts from atmospheric pressure, and is subsequently pumped to ≈3 mbar, which takes on the order of the time necessary for the drop to freeze; the jet is directly made at 3 mbar and therefore evaporates faster.

In the first deposition technique, we employ a traditional layer-by-layer strategy. The printhead translates continuously in the horizontal plane, depositing water droplets that spread and freeze almost instantly after impact to form solid layers. By sequentially stacking these horizontal layers on top of one another, the system builds up volumetric 3D geometries from the bottom up.

**Fig. 3.**
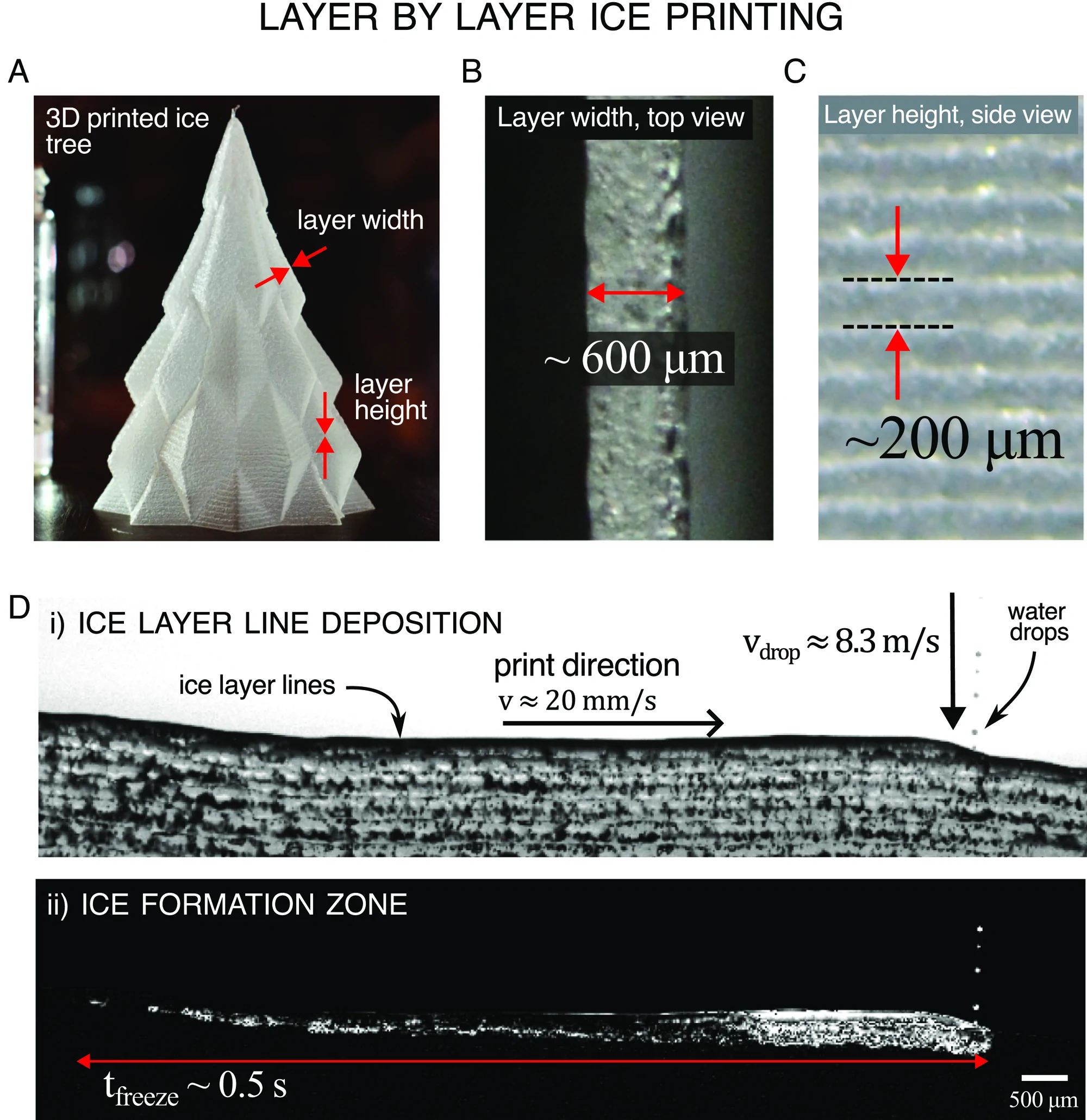
(*A*) Example of a layer-by-layer 3D printed ice tree. The ice layer height and wall thickness depend on both the print speed and the jet flow rate. For one representative setting, the wall thickness was measured to be approximately 600 μm (*B*, top view of a printed wall) and the layer height 200 μm (*C*, side view of a printed wall). (*D*) High-speed imaging snapshot showing the deposition of micro droplets onto previously printed ice layer lines (side view). The droplet impact velocity (≈8.3 m s^−1^) is significantly higher than the printer’s travel speed (≈20 mm s^−1^).The *Upper* subpanel (*i*) shows the raw high-speed image, while the *Lower* subpanel (*ii*) shows the same frame after subtraction of an earlier image, revealing the ice formation zone. This indicates that the newly deposited line takes approximately 0.5 s to fully freeze.

In contrast to conventional fused deposition modeling (FDM), the layer line width and layer height of the ice structures are not directly related to the nozzle diameter. In fact, as the velocity of impacting droplets is much higher than the printer’s traveling speed, droplets accumulate at the region of deposition. The dimensions of the printed layer lines are therefore a complex interplay between the wetting of water on ice, the evaporative cooling rate, the extrusion rate (i.e., liquid flow rate), and the printhead travel speed. For the used printer settings, such as printing an 3D ice tree (Fig. 3*A*), we measured the layer line characteristics by means of image analysis using ImageJ and a Zeiss microscope slide scale bar for reference. Fig. 3*B*, shows a top view of a printed ice wall, with a layer line width of ∼600 μm. A side view, Fig. 3*C*, clearly shows the individual layers, indicating that the freezing kinetics allowed for shape retention. The measured layer height of ∼200 μm corresponds to the vertical resolution of the current printing settings. Note that for this first demonstration, the nozzle size was chosen for robustness and simplicity; employing a printing system with smaller nozzles, higher mechanical stability and faster translation speeds should enable the fabrication of even thinner structures.

In agreement with earlier scaling-based estimates, high-speed imaging at 110,000 fps (Fig. 3*D*) reveals that drops formed from the jet arrive as liquid water on the printed structure and subsequently freeze within about 0.5 s. The measurements show that $v_{\text{drop}}\approx 8.3$ m s^−1^, confirming that droplets impact the substrate far faster than the lateral printhead motion described above. Under the present operating conditions—constant jet flow rate and a relatively slow translation speed—this velocity mismatch leads to the accumulation of multiple droplets on top of the previously printed ice layer. Consequently, the structural accuracy of the print relies strongly on the partial wetting behavior of liquid water on ice. As established in recent experimental studies, water maintains a finite macroscopic contact angle on ice rather than spreading into a thin film (16–19). This restricted spreading is critical for the mechanics of the 3D printing process; it ensures that the accumulated liquid forms a distinct, stable layer that retains its intended shape before freezing, rather than flowing down the sides of the structure. A direct observation of this freezing process is provided by image subtraction, which visualizes the ice formation zone in the *Lower* panel of Fig. 3*D*. The subtraction suppresses static features (shown in black) and highlights regions where change occurs. As indicated by the red arrow, this liquid zone spans approximately 10 mm.

**Fig. 4.**
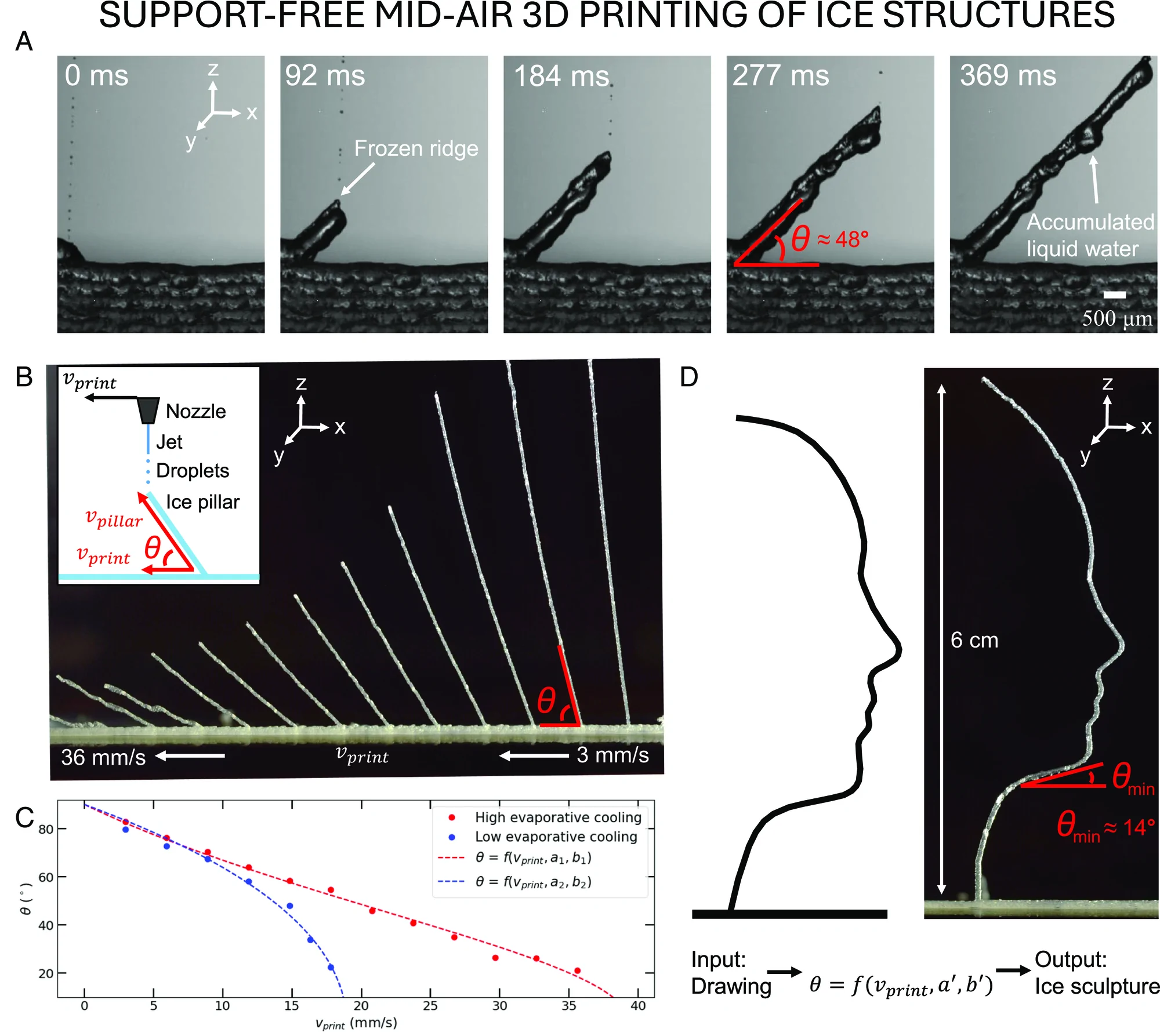
(*A*) High-speed snapshots of a free-standing ice pillar being printed as the horizontal print velocity is reduced. A frozen ridge forms along the top, generating vertical lift-off. As shown, liquid water temporarily accumulates underneath this frozen ridge before completely freezing. (*B*) Free-standing ice pillars printed at varying velocities, demonstrating the relationship between nozzle speed and structure inclination. The printing direction is from right to left. As the print velocity ($v_{\text{print}}$) increases from 3 mm s^−1^ on the right to 36 mm s^−1^ on the left, the resulting printing angle (θ) decreases, causing the pillars to lean closer to the horizontal. (*Inset*) Schematic of the in vacuo ice printing setup, illustrating the geometric relationship between the horizontal print velocity ($v_{\text{print}}$), the pillar growth velocity ($v_{\text{pillar}}$), and the resulting angle (θ). The full printing process is captured in the Movie S3. (*C*) Quantitative relationship between the resulting printing angle (θ) and the print velocity ($v_{\text{print}}$) under two distinct thermodynamic conditions. The experimental data reveal a divergence in structural behavior between high (red circles) and low (blue circles) evaporative cooling environments. The dashed lines represent two-parameter mathematical fits, $\theta =f(v_{\text{print}},a,b)$, applied to each dataset. The data obtained under high evaporative cooling conditions (red circles) correspond directly to the printed pillars displayed in *B*. (*D*) Demonstration of support-free, mid-air ice sculpting utilizing the established velocity-to-angle relationship. By translating an input drawing (*Left*) into a continuous sequence of required local angles, the corresponding print velocities are calculated using the empirical fit $\theta =f(v_{\text{print}},a^{\prime },b^{\prime })$. This allows the system to dynamically modulate the print speed to construct complex and continuous profiles. The human face shown on the right demonstrates this capability, having been printed entirely without auxiliary support structures. The physical limit of this support-free overhang capability is highlighted at the neck region, where the minimum achievable printing angle reaches $\theta_{\text{min}}\approx 14^{{}^{\circ}}$. The 6-cm-tall sculpture was printed in 8 s. The mid-air printing process of the sculpture is captured in the Movie S4.

The second deposition technique departs from these planar constraints to enable support-free mid-air printing. By deliberately reducing the horizontal translation speed of the printhead relative to the extrusion rate, the water is forced to accumulate and freeze upward. This rapid localized freezing generates immediate vertical lift-off, allowing for the continuous extrusion of free-standing, out-of-plane structures directly into empty space without the need for any underlying substrate or scaffolding. The simplest example of such a printing mode is to keep the nozzle at a fixed position, which leads to the formation of a vertical ice pillar. By also slowly moving the print head sideways, ice pillars with different angles can be fabricated. Fig. 4*A* shows high speed images demonstrating this mechanism. As can be seen, a thin climbing ice ridge forms, while still-unfrozen water accumulates beneath it before freezing, forming an ice strut with an angle of 48°. To quantitatively understand and harness the geometric capabilities of this mid-air printing process, we systematically investigated the relationship between the horizontal print velocity ($v_{\mathrm{print}}$) and the resulting inclination angle (θ) of the printed ice pillar, as shown in Fig. 4*B*. Ice pillars were printed with varying velocities from 3 to 36 mm s^−1^. With increasing print velocity, the inclination of the pillars decreases. The exact inclination angle, however, is highly sensitive to the ambient thermodynamic conditions. In particular, we observed faster ice formation in a predried vacuum chamber and when printing at an elevation aligned with the chamber’s vacuum inlet. Fig. 4*C* shows the empirical relationship between θ and $v_{\mathrm{print}}$ under high and low evaporative cooling regimes. The key difference between the two experiments is the initial relative humidity (RH) in the vacuum chamber prior to evacuation. The high evaporative cooling regime starts with a RH of ∼10%, while the low evaporative cooling regime begins at a RH of ∼35%. For both conditions, the angular response can be accurately described by a two-parameter mathematical fit ($\theta =f(v_{\mathrm{print}},a,b)$), where

[2] $$\begin{array}{c} f\left(v_{\mathrm{print}},a,b\right)=\;\arccos\left(\frac{v_{\mathrm{print}}}{a\;\cdot \;v_{\mathrm{print}}\;+\;b}\right). \end{array}$$

In this model, the denominator represents a first-order linear approximation of the pillar growth velocity ($v_{\mathrm{pillar}}$). As $v_{\mathrm{print}}$ increases and the deposition angle becomes more horizontal, water tends to accumulate in localized pockets beneath the frozen ridge (Fig. 4*A*). While the formation of these pockets increases $v_{\mathrm{pillar}}$ on average, it yields a noticeably rougher and less structurally controlled strut.

By precisely controlling this velocity–angle relationship, we can reliably print structures at varying angles, demonstrating that the ice maintains sufficient mechanical strength to support its own weight. Moreover, these structures demonstrated remarkable flexibility: when the printer was shaken, the slender ice struts oscillated with large amplitudes without breaking (*SI Appendix*). The ability to realize such delicate, unsupported geometries opens the door to creating complex negative molds. For example, printed spiral or branching ice structures can be used as sacrificial templates: After casting a material around them, the ice is simply melted away to reveal hollow channels, such as artificial arteries or microfluidic devices (1, 3).

The predictability of this velocity-to-angle relationship, for a given experimental setup, also unlocks a powerful capability: the direct, support-free fabrication of complex, mid-air 3D architectures. By utilizing the established empirical fit, we can invert the problem—translating an input drawing into a sequence of required local angles to calculate the specific, dynamically modulated print velocities needed along a continuous path. Fig. 4*D* illustrates this principle through the mid-air sculpting of a human face in profile. The system continuously adjusts $v_{\mathrm{print}}$ to trace the intricate curves of the design. This technique enables the direct fabrication of such stable overhangs without auxiliary support structures, overcoming a common limitation in conventional additive manufacturing. In this example, the printed ice strut reaches an inclination of $\theta \approx 14^{{}^{\circ}}$ at the neck of the sculpture, demonstrating the ability to fabricate unsupported structures at angles approaching horizontal.

In summary, we have demonstrated 3D ice-printing methods that differ from existing techniques, which typically rely on cooled substrates or subzero ambient temperatures, both involving substantial cooling infrastructure (4). Our vacuum evaporative approach requires no dedicated cryogenic infrastructure. Instead it relies on readily available vacuum and 3D-printing components, thus keeping operating costs well below those of liquid-nitrogen- or helium-based systems. The method exploits the thermodynamic instability of liquid water at low pressure, where rapid evaporation extracts latent heat and cools the extruded water far below 0 °C. The printed structures consist of ice without supporting material, structural additives, or foreign particles, which is crucial for applications in microfluidics and tissue engineering where material purity directly affects biocompatibility and fluid properties. The only additive used was a trace amount of NaCl in the printing liquid, which was added to suppress possible electrostatic charging effects during deposition. Interestingly, the thin printed ice structures exhibit remarkable flexibility: long, slender struts were observed to vibrate with large amplitudes without breaking. This suggests that tailored geometries could be used to realize ice structures with mechanical responses very different from those of bulk ice, opening opportunities for both functional and aesthetic applications.

## 2. Discussion

Our technique is intrinsically scalable: independent control over jet diameter, flow rate, chamber pressure, and printing speed allows both resolution and production rate to be tuned as needed. Furthermore, the output is exceptionally clean; once the chamber is vented, the printed ice melts back to liquid water without leaving any residue, making it straightforward to integrate the process with downstream fabrication or processing steps.

From a materials perspective, ice-templated structures have long been used to create hierarchical porous materials (3, 20, 21). Freeze-casting, where suspensions are directionally frozen and then dried, produces anisotropic porous ceramics and composites with tailored pore structure and mechanical properties. Our method offers a complementary approach: Instead of freezing a suspension in a mold, we sculpt ice directly, which can then serve as a sacrificial template for microfluidic channel networks (22), tissue scaffolds (23), or complex casting forms.

Beyond the immediate application of 3D ice printing, the rapid evaporative freezing dynamics characterized in this study offer a highly controlled, macroscopic analog for atmospheric ice nucleation. Ice formation in clouds occurs via nucleation of water droplets in the upper troposphere, where pressures and temperatures are low. Pure liquid droplets can spontaneously freeze if the cooling is rapid enough (24). Our macroscopic demonstration provides a conceptual model for understanding nucleation at the microscale.

Several extensions of this work are foreseeable:

*Bridging:* For many 3D printing techniques, bridging presents a major challenge in fabricating complex structures. Here, we demonstrate that, beyond conventional layer-by-layer deposition, curved, angled, and straight ice pillars can be formed simply by reducing the print velocity while maintaining the same jet velocity. This principle can be exploited to create support elements or bridged structures, and greater control over this mechanism could prove invaluable for future applications.
*Hybrid printing:* Water could be mixed with dissolved polymers, salts, or nanoparticles before injection, allowing the deposition of composite ice–material structures. Upon melting or selective sublimation, these could yield polymer or inorganic scaffolds with controlled geometry.
*Layer line smoothing:* Layer-line smoothing in FDM printing is commonly used to improve the visual appearance of printed objects. For ice, this process would be particularly simple: partially melting and refreezing the surface of the printed object would result in a smoothened ice print.
*Planetary applications:* The Martian polar regions contain water ice and operate under near-vacuum conditions (pressures ~1 to 10 mbar, temperatures ~−100 to −150 °C). Our method could potentially be adapted for ISRU, using Martian water to construct habitats, thermal shielding, or radiation barriers without importing external materials or refrigeration systems.
*Tissue engineering and regenerative medicine:* Pure ice scaffolds with controlled pore size and interconnectivity can be used as temporary supports for cell seeding, vascularization, and tissue growth. Upon melting, the scaffold dissolves without chemical residue, leaving behind organized tissue.
*Current printing limitations:* As a standard 3D printer was used, the printing accuracy is primarily limited by mechanical factors such as backlash, vibrations, and dimensional tolerances. Moreover, the jet velocity is much higher than the printer’s travel speed, causing droplets to accumulate at their landing position while the print head moves. This results in walls that are significantly thicker than the jet diameter, with the final wall thickness mainly determined by the wetting properties of water on ice. Although smaller jets could easily be implemented, our current system lacks the positional accuracy required to take advantage of them. A more advanced motion system, capable of high-speed and highly precise positioning, could enable an entirely different printing regime–true droplet-by-droplet deposition–potentially facilitated by stimulated jet breakup to produce a monodisperse droplet train. These higher printing resolutions could reach submillimeter scales, enabling intricate microarchitected ice structures for microfluidic and photonic applications.

## 3. Conclusion

In sum, we have demonstrated a simple, elegant, and cryogen-free method for three-dimensional printing of ice structures via evaporative cooling in vacuum. A commercial 3D printer, modified with a fine water jet nozzle fed by an HPLC pump and housed in an acrylic vacuum chamber, produces complex ice geometries with high fidelity. The underlying physics rests on the thermodynamic instability of liquid water at low pressure, where rapid evaporation extracts latent heat and cools the extruded water far below 0 °C.

The method requires no cryogenic infrastructure, no supporting material, and no external refrigeration, making it accessible to many laboratories and enabling applications in microfluidics, tissue engineering, and materials templating. The visual demonstration of evaporative cooling and phase transitions also serves as a powerful educational tool.

This work opens a frontier in additive manufacturing of ice and demonstrates the power of exploiting simple thermodynamic principles for engineering innovation. Future directions include miniaturization for high-resolution microstructures, hybrid printing with dissolved additives, and adaptation to planetary environments such as those found on the moon.

## 4. Materials and Methods

**Fig. 5.**
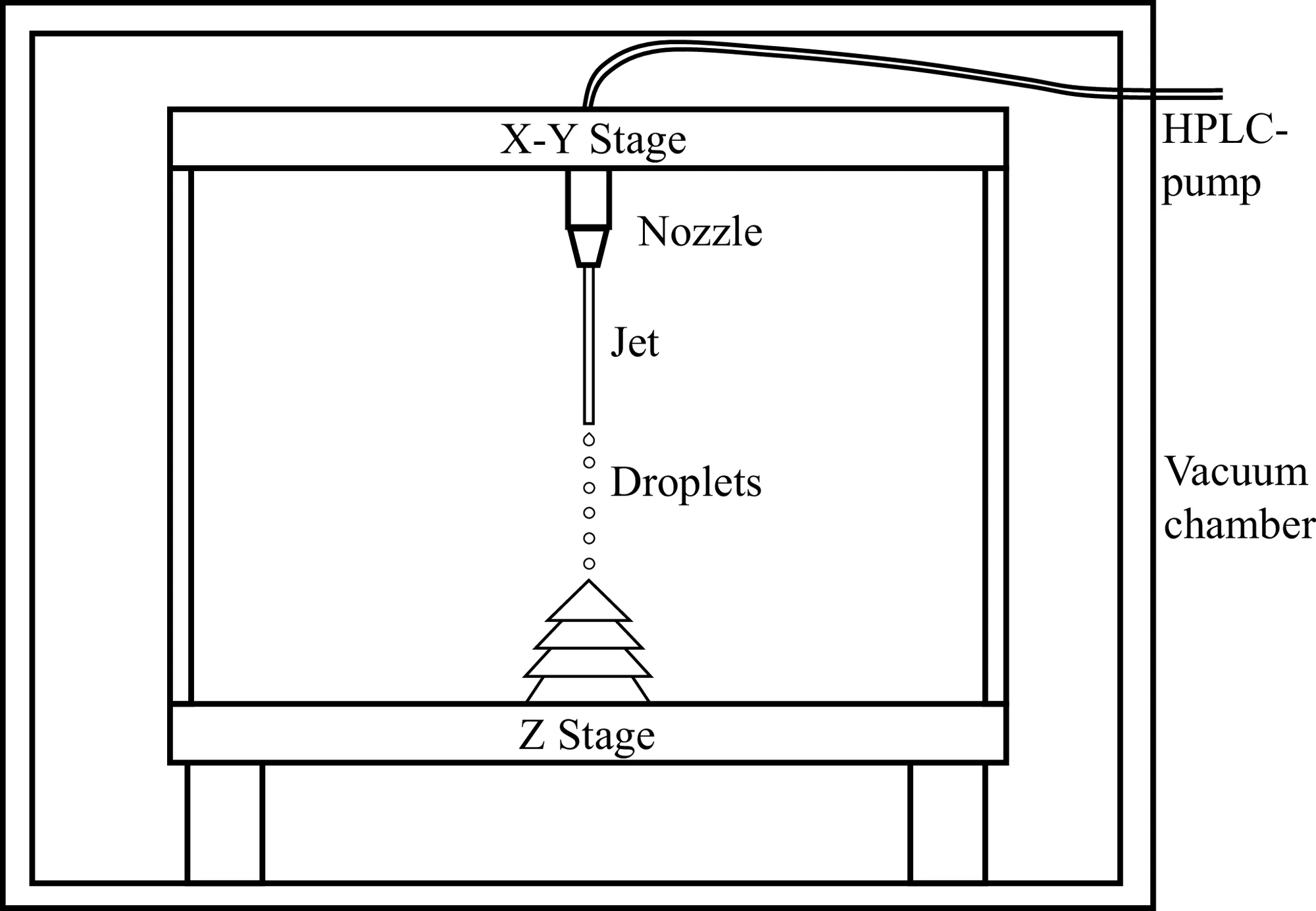
Schematic representation of the 3D ice printing setup. A vacuum chamber houses a 3D positioning system where a nozzle, driven by an high-performance liquid chromatography (HPLC) pump, ejects a 16 μm wide water jet. The jet breaks into droplets that freeze onto the substrate via evaporative cooling to form a solid structure.

Our setup consists of a commercial 3D printer (ROOK MK1, motion control via open-source firmware) housed inside a transparent acrylic vacuum chamber (Sanatron, inner dimensions: 0.38 m × 0.36 m × 0.37 m), as shown in Fig. 5. The standard thermoplastic extruder of the 3D printer was replaced with a custom nozzle mount designed to hold a standard PEK Luer-lock HPLC-adapter. Water is supplied by an HPLC pump, connected to the nozzle via capillary tubing. A 0.45 μm syringe filter can be used to dampen pressure fluctuations during the pump cycles, which otherwise manifest as periodically appearing thicker layer lines in the print (see Fig. 3*A* as an example without filter). The nozzle consists of a microfabricated Si3N4 chip with a 16 μm diameter orifice (Medspray), produced by photolithography and mounted on a polypropylene adapter, which generates a stable laminar water jet with a typical velocity of v∼8 m/s. While the present implementation uses an HPLC pump and a microfabricated nozzle chip, these components are not essential to the underlying concept of vacuum-assisted ice printing and could be replaced by alternative liquid delivery and nozzle systems in future implementations. The vacuum chamber is evacuated using a rotary vane pump (Oerlikon Leybold SOGEVAC SV 16 D) and the chamber pressure is monitored continuously using an analogue manometer and kept at around 2 to 3 mbar during printing runs. The heated bed was replaced by a laser-cut plastic plate, the surface of which was lightly sanded to facilitate ice nucleation and crystallization. The print bed is not actively cooled, as the evaporative cooling is sufficient to maintain the print object at freezing temperature. However, a precooled or actively cooled print bed could improve adhesion of the printed object to the bed. Distilled water containing a trace amount of NaCl (>1 × 10^−4^ M) was used to increase the electrical conductivity of the liquid and prevent electro static self-charging of the droplets during printing. This effect can be overlooked when working with highly purified, low-conductivity water, but may lead to artifacts in droplet placement and deposition stability in a nonconductive environment (25).

## Supplementary Material

Appendix 01 (PDF)

Dataset S01 (XLSX)

Movie S1. Timelapse video of the 3D-printed ice Christmas tree (similar to the structure presented in Fig. 1 of the main article). The object was printed layer-by-layer at a chamber pressure of 2 mbar. The actual printing process took 26 minutes to reach a final height of approximately 8 cm.

Movie S2. Timelapse video of the melting process of the 3D-printed ice Christmas tree. Melting was induced by releasing the vacuum chamber back to an ambient pressure of 1 bar. The complete melting process took approximately 13 minutes, roughly half the time required to print the structure.

Movie S3. Video of the continuous printing process for free-standing ice pillars at varying velocities (corresponding to the structure presented in Fig. 4b of the main article). The printing proceeds from left to right. As the horizontal print velocity decreases from 36 mm/s to 3 mm/s, the video demonstrates the direct relationship between nozzle speed and structure inclination, with the printing angle (*θ*) progressively increasing. The movie is sped up 2 times.

Movie S4. Real-time video demonstrating the support-free, mid-air printing of a continuous ice sculpture (human face profile, corresponding to Fig. 4d). The video illustrates the dynamic modulation of the print velocity to construct varying local angles based on the empirical relationship *θ* = f (*v_print_*,*a’*,*b’*). The entire 6-cm-tall structure is printed without auxiliary supports in 8 seconds.

## Data Availability

All study data are included in the article and/or supporting information.
