# Supplementary material for: Three-dimensional printing of ice structures via evaporative cooling in vacuum
Source: Proc Natl Acad Sci U S A. 2026 Sep 2;123(36):e2608173123. doi: 10.1073/pnas.2608173123 (PMC13552893; doi:10.1073/pnas.2608173123)
Supplement: Supplementary file 1 [file pnas.2608173123.sapp.pdf]

Three-Dimensional Printing of Ice Structures via Evaporative Cooling in Vacuum

Menno Demmenie,[*,†,‡] Stefan Kooij,[†,‡] and Daniel Bonn[†]

†*Institute of Physics, University of Amsterdam, Science Park 904, 1098 XH Amsterdam, the Netherlands*

‡*These authors contributed equally to this work.*

E-mail: m.demmenie@uva.nl*

Supplemental Material

Flexibility of printed ice pillar

We performed additional experiments in which ice pillars were grown up to the nozzle head and subsequently released by laterally moving the printer head, causing the pillars to bend and eventually break loose from the nozzle. After release, the pillars exhibited a damped oscillatory motion, demonstrating the flexibility of the structure as was mentioned before in the paper. These oscillations were recorded and analysed (Fig. S1a) and can be well described by a simple damped harmonic oscillator model, yielding an oscillation frequency of ∼8.6 Hertz for an ice pillar of 8 cm. Figure S1b shows a representative ice pillar (i) together with a maximum-intensity projection of the recorded oscillation sequence (ii), illustrating the spatial variation of the oscillation amplitude along the length of the strut.

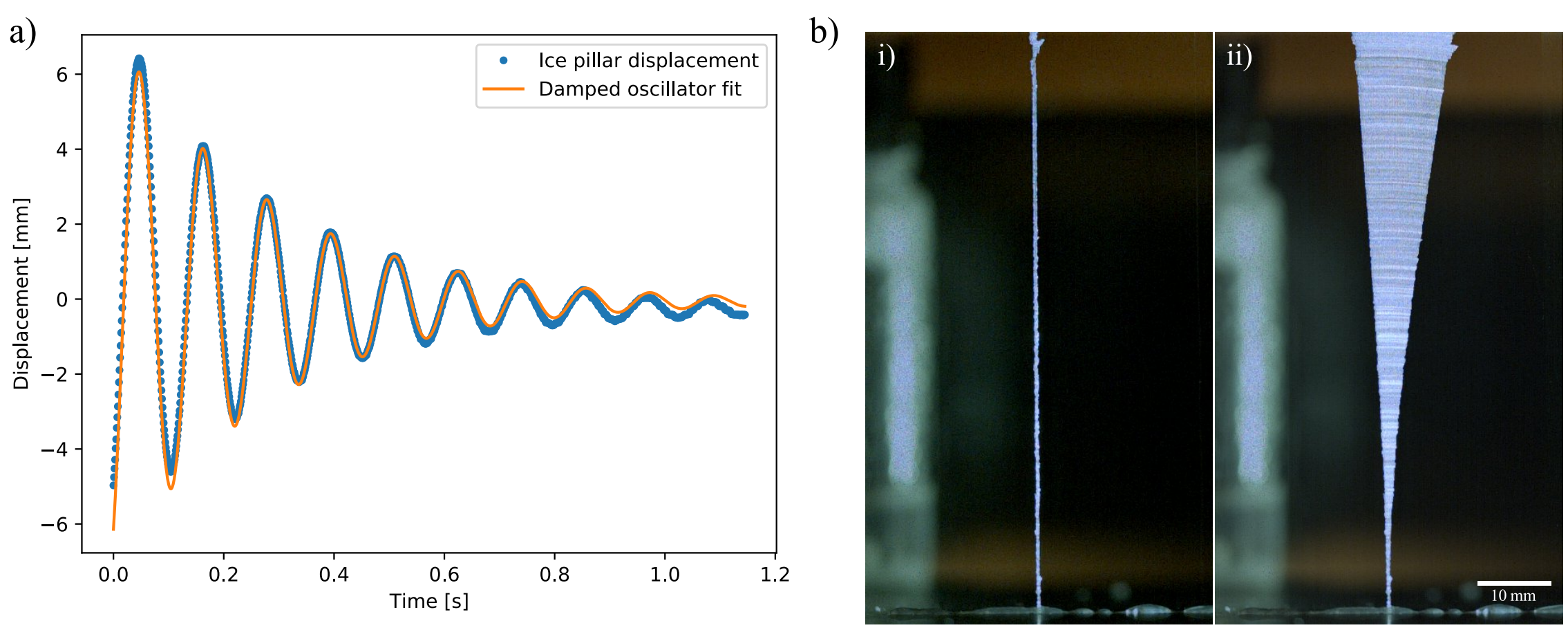

Figure S1: a) Measured oscillation amplitude of an ice strut as a function of time. The displacement data are fitted with a damped harmonic oscillator, yielding an oscillation frequency of 8.64 Hertz. b) Image of the investigated ice strut (i) and the summed maximum-intensity over all oscillation frames (ii), showing the maximum oscillation amplitude along the length of the strut.

Description of supplementary movie files

Movie S1

Timelapse video of the 3D-printed ice Christmas tree (similar to the structure presented in Fig. 1 of the main article). The object was printed layer-by-layer at a chamber pressure of 2 mbar. The actual printing process took 26 minutes to reach a final height of approximately 8 cm.

Movie S2

Timelapse video of the melting process of the 3D-printed ice Christmas tree. Melting was induced by releasing the vacuum chamber back to an ambient pressure of 1 bar. The complete melting process took approximately 13 minutes, roughly half the time required to print the structure.

Movie S3

Video of the continuous printing process for free-standing ice pillars at varying velocities (corresponding to the structure presented in Fig. 4b of the main article). The printing proceeds from left to right. As the horizontal print velocity decreases from 36 mm/s to 3 mm/s, the video demonstrates the direct relationship between nozzle speed and structure inclination, with the printing angle (θ) progressively increasing. The movie is sped up 2 times.

Movie S4

Real-time video demonstrating the support-free, mid-air printing of a continuous ice sculpture (human face profile, corresponding to Fig. 4d). The video illustrates the dynamic modulation of the print velocity to construct varying local angles based on the empirical relationship $\theta = f(v_{print}, a', b')$. The entire 6-cm-tall structure is printed without auxiliary supports in 8 seconds.
